# *catena*-Poly[nickel(II)-bis­(μ-2-amino­ethane­sulfonato-κ^3^ *N*,*O*:*O*′;κ^3^ *O*:*N*,*O*′)]

**DOI:** 10.1107/S1600536810020325

**Published:** 2010-06-05

**Authors:** Feng Yang, Zhi-Hong Wu, Jin-Hua Cai

**Affiliations:** a Key Laboratory for the Chemistry and Molecular Engineering of Medicinal Resources (Ministry Education of China), School of Chemistry & Chemical Engineering, Guangxi Normal University, Guilin 541004, People’s Republic of China; b Department of Chemistry and Life Science, Hechi University, Yizhou, Guangxi 546300, People’s Republic of China

## Abstract

In the title polymeric complex, [Ni(C_2_H_6_NO_3_S)_2_]_*n*_, the Ni^II^ ion occupies a special position on an inversion centre and displays a slightly distorted octa­hedral coordination geometry, being linked to four sulfonate O atoms and to two N atoms of the taurine ligands. The sulfonate groups doubly bridge symmetry-related Ni^II^ centers, forming polymeric chains along the *a* axis.

## Related literature

For general background to taurine complexes and their derivatives, see: Bottari & Festa (1998 ▶); Zhang & Jiang (2002 ▶); Zeng *et al.* (2003 ▶); Zhong *et al.* (2003 ▶). For our previous work on taurine complexes, see: Cai *et al.* (2004 ▶, 2006 ▶); Jiang *et al.* (2005 ▶).
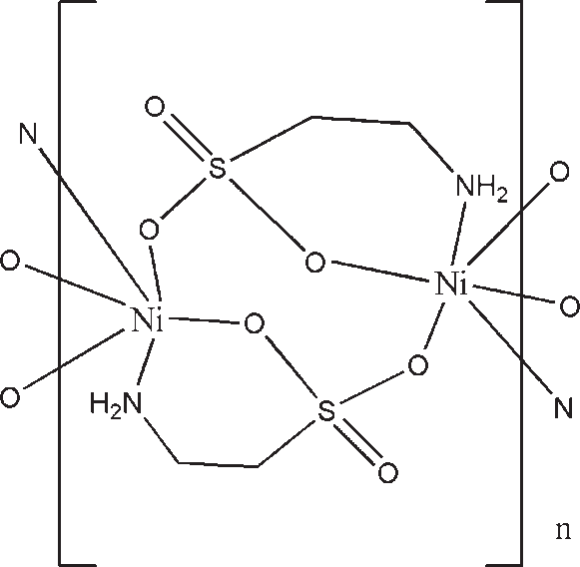


## Experimental

### 

#### Crystal data

[Ni(C_2_H_6_NO_3_S)_2_]
*M* *_r_* = 306.99
Monoclinic, 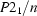

*a* = 5.1003 (17) Å
*b* = 8.231 (3) Å
*c* = 11.673 (4) Å
β = 97.492 (4)°
*V* = 485.9 (3) Å^3^
*Z* = 2
Mo *K*α radiation
μ = 2.44 mm^−1^
*T* = 293 K
0.20 × 0.16 × 0.08 mm

#### Data collection

Bruker SMART APEX CCD area-detector diffractometer
Absorption correction: multi-scan (*SADABS*; Bruker, 1999 ▶) *T*_min_ = 0.632, *T*_max_ = 0.829
2116 measured reflections
956 independent reflections
881 reflections with *I* > 2σ(*I*)
*R*_int_ = 0.026

#### Refinement

*R*[*F* ^2^ > 2σ(*F* ^2^)] = 0.027
*wR*(*F* ^2^) = 0.072
*S* = 1.06
954 reflections
76 parameters
H atoms treated by a mixture of independent and constrained refinement
Δρ_max_ = 0.44 e Å^−3^
Δρ_min_ = −0.43 e Å^−3^

### 

Data collection: *SMART* (Bruker, 1999 ▶); cell refinement: *SAINT* (Bruker, 1999 ▶); data reduction: *SAINT*; program(s) used to solve structure: *SHELXS97* (Sheldrick, 2008 ▶); program(s) used to refine structure: *SHELXL97* (Sheldrick, 2008 ▶); molecular graphics: *SHELXTL* (Sheldrick, 2008 ▶); software used to prepare material for publication: *SHELXTL*.

## Supplementary Material

Crystal structure: contains datablocks I, global. DOI: 10.1107/S1600536810020325/bh2285sup1.cif

Structure factors: contains datablocks I. DOI: 10.1107/S1600536810020325/bh2285Isup2.hkl

Additional supplementary materials:  crystallographic information; 3D view; checkCIF report

## Figures and Tables

**Table 1:** Selected bond lengths (Å)

Ni1—N1^i^	2.054 (2)
Ni1—N1^ii^	2.054 (2)
Ni1—O1^ii^	2.0916 (17)
Ni1—O1^i^	2.0916 (17)
Ni1—O2	2.1185 (18)
Ni1—O2^iii^	2.1185 (18)

Symmetry codes: (i) 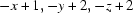; (ii) 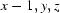; (iii) 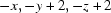.

**Fig. 1.**
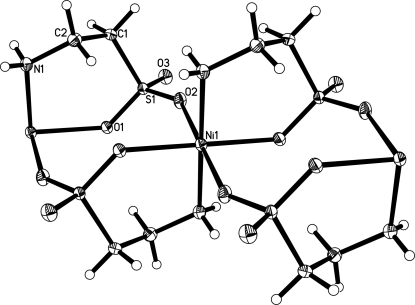
A segment of the polymeric structure of (I) with 30% probability displacement ellipsoids (arbitrary spheres for H atoms)

**Fig. 2.**
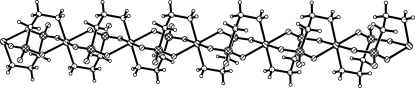
The one-dimensional polymeric chain of the title complex.

## supplementary crystallographic information

### Comment

Taurine, an amino acid containing sulfur, is indispensable to human beings because of its applications in medicine and biochemistry (Bottari & Festa, 1998; Zhang & Jiang, 2002; Zeng *et al.*, 2003; Zhong *et al.*, 2003). Several taurine complexes and their derivatives have recently been prepared in our laboratory (Cai *et al.*, 2004; Jiang *et al.*, 2005; Cai *et al.*, 2006). As part of our ongoing investigation, the title polymeric Ni^II^ complex, (I), has been prepared and its structure determined.

A segment of the polymeric structure of (I) is illustrated in Fig. 1. The Ni^II^ ion is coordinated by four sulfonate O atoms and to two N atoms of the taurine ligands, displaying distorted octahedral coordination geometry. The sulfonate anions act as bridging ligands in (I). Neighbouring Ni atoms are bridged by two sulfonate anions, to form a zigzag polymeric chain along the *a* axis, as shown in Fig. 2. The polymeric chain has a repeat unit formed by two taurine and two Ni^II^ atoms related by an inversion centre, which coincides with the centre of the eight-membered Ni_2_S_2_O_4_ ring formed by the atoms of two bridging ligands and the Ni atoms; the distance between the two Ni atoms is 5.100 (12) Å. In the structure of the title compound, there are two symmetry-independent "active" H atoms; both of them belong to the NH_2_ group of the taurine ligand. They form intramolecular hydrogen bonds with sulfonate atom O3.

### Experimental

A solution of taurine (1.0 mmol) and KOH (1.0 mmol) in anhydrous methanol (10 ml) was added slowly to a solution of Ni(CH_3_COO)_2_ (1.0 mmol) in anhydrous methanol (10 ml). After stirring for 10 min, it was then dropped into a 25 ml Teflon-lined stainless steel reactor and heated at 393 K for five days. Thereafter, the reactor was slowly cooled to room temperature and green block-shaped crystals suitable for X-ray diffraction were collected.

### Refinement

H atoms were positioned geometrically (C—H = 0.97 Å and N—H = 0.80 Å) and included in the refinement in the riding-model approximation, with *U*_iso_(H) = 1.2*U*_eq_(carrier atom).

### Crystal data

**Table:**

[Ni(C_2_H_6_NO_3_S)_2_]	*F*(000) = 316
*M**_r_* = 306.99	*D*_x_ = 2.098 Mg m^−^^3^
Monoclinic, *P*2_1_/*n*	Mo *K*α radiation, λ = 0.71073 Å
Hall symbol: -P 2yn	Cell parameters from 783 reflections
*a* = 5.1003 (17) Å	θ = 3.0–27.6°
*b* = 8.231 (3) Å	µ = 2.44 mm^−^^1^
*c* = 11.673 (4) Å	*T* = 293 K
β = 97.492 (4)°	Block, green
*V* = 485.9 (3) Å^3^	0.20 × 0.16 × 0.08 mm
*Z* = 2	

### Data collection

**Table:**

Bruker SMART APEX CCD area-detector diffractometer	956 independent reflections
Radiation source: fine-focus sealed tube	881 reflections with *I* > 2σ(*I*)
graphite	*R*_int_ = 0.026
φ and ω scans	θ_max_ = 26.0°, θ_min_ = 3.0°
Absorption correction: multi-scan (*SADABS*; Bruker, 1999)	*h* = −5→6
*T*_min_ = 0.632, *T*_max_ = 0.829	*k* = −6→10
2116 measured reflections	*l* = −14→14

### Refinement

**Table:**

Refinement on *F*^2^	Primary atom site location: structure-invariant direct methods
Least-squares matrix: full	Secondary atom site location: difference Fourier map
*R*[*F*^2^ > 2σ(*F*^2^)] = 0.027	Hydrogen site location: inferred from neighbouring sites
*wR*(*F*^2^) = 0.072	H atoms treated by a mixture of independent and constrained refinement
*S* = 1.06	*w* = 1/[σ^2^(*F*_o_^2^) + (0.044*P*)^2^ + 0.1*P*] where *P* = (*F*_o_^2^ + 2*F*_c_^2^)/3
954 reflections	(Δ/σ)_max_ = 0.001
76 parameters	Δρ_max_ = 0.44 e Å^−^^3^
0 restraints	Δρ_min_ = −0.43 e Å^−^^3^
0 constraints	

### Fractional atomic coordinates and isotropic or equivalent isotropic displacement parameters (Å^2^)

**Table:**

	*x*	*y*	*z*	*U*_iso_*/*U*_eq_	
Ni1	0.0000	1.0000	1.0000	0.01738 (17)	
S1	0.46761 (11)	0.95864 (7)	0.81432 (5)	0.01601 (18)	
O1	0.6637 (3)	1.0584 (2)	0.88498 (15)	0.0213 (4)	
O2	0.2125 (3)	0.9622 (2)	0.85798 (16)	0.0241 (4)	
O3	0.4412 (4)	1.0004 (2)	0.69297 (16)	0.0255 (4)	
C1	0.5831 (5)	0.7569 (3)	0.8243 (2)	0.0228 (5)	
H1A	0.4468	0.6865	0.7857	0.027*	
H1B	0.7363	0.7484	0.7833	0.027*	
C2	0.6583 (4)	0.6964 (3)	0.9465 (2)	0.0222 (5)	
H2A	0.5292	0.7340	0.9946	0.027*	
H2B	0.6568	0.5785	0.9469	0.027*	
N1	0.9230 (4)	0.7550 (3)	0.99449 (19)	0.0196 (4)	
H1C	0.963 (6)	0.719 (4)	1.058 (3)	0.024*	
H1D	1.023 (6)	0.715 (4)	0.956 (3)	0.024*	

### Atomic displacement parameters (Å^2^)

**Table:**

	*U*^11^	*U*^22^	*U*^33^	*U*^12^	*U*^13^	*U*^23^
Ni1	0.0148 (2)	0.0200 (3)	0.0172 (3)	−0.00114 (15)	0.00144 (18)	−0.00013 (16)
S1	0.0137 (3)	0.0212 (3)	0.0132 (3)	0.0001 (2)	0.0022 (2)	−0.0009 (2)
O1	0.0194 (8)	0.0201 (9)	0.0230 (9)	−0.0006 (7)	−0.0025 (7)	−0.0012 (7)
O2	0.0156 (8)	0.0361 (10)	0.0216 (10)	−0.0001 (7)	0.0062 (7)	0.0006 (7)
O3	0.0274 (10)	0.0341 (11)	0.0153 (10)	−0.0008 (7)	0.0038 (8)	0.0021 (7)
C1	0.0224 (12)	0.0205 (12)	0.0243 (13)	0.0017 (10)	−0.0015 (10)	−0.0071 (10)
C2	0.0196 (11)	0.0190 (11)	0.0287 (13)	−0.0028 (9)	0.0060 (10)	0.0014 (10)
N1	0.0204 (10)	0.0213 (10)	0.0171 (10)	0.0001 (9)	0.0018 (8)	0.0032 (9)

### Geometric parameters (Å, °)

**Table:**

Ni1—N1^i^	2.054 (2)	O1—Ni1^iv^	2.0916 (17)
Ni1—N1^ii^	2.054 (2)	C1—C2	1.513 (3)
Ni1—O1^ii^	2.0916 (17)	C1—H1A	0.9700
Ni1—O1^i^	2.0916 (17)	C1—H1B	0.9700
Ni1—O2	2.1185 (18)	C2—N1	1.474 (3)
Ni1—O2^iii^	2.1185 (18)	C2—H2A	0.9700
S1—O3	1.447 (2)	C2—H2B	0.9700
S1—O2	1.4584 (18)	N1—Ni1^iv^	2.054 (2)
S1—O1	1.4630 (18)	N1—H1C	0.80 (3)
S1—C1	1.760 (2)	N1—H1D	0.80 (3)
			
N1^i^—Ni1—N1^ii^	180.000 (1)	S1—O1—Ni1^iv^	132.53 (11)
N1^i^—Ni1—O1^ii^	86.09 (8)	S1—O2—Ni1	147.91 (12)
N1^ii^—Ni1—O1^ii^	93.91 (8)	C2—C1—S1	114.49 (17)
N1^i^—Ni1—O1^i^	93.91 (8)	C2—C1—H1A	108.6
N1^ii^—Ni1—O1^i^	86.09 (8)	S1—C1—H1A	108.6
O1^ii^—Ni1—O1^i^	180.000 (1)	C2—C1—H1B	108.6
N1^i^—Ni1—O2	93.06 (8)	S1—C1—H1B	108.6
N1^ii^—Ni1—O2	86.94 (8)	H1A—C1—H1B	107.6
O1^ii^—Ni1—O2	89.52 (7)	N1—C2—C1	110.97 (19)
O1^i^—Ni1—O2	90.48 (7)	N1—C2—H2A	109.4
N1^i^—Ni1—O2^iii^	86.94 (8)	C1—C2—H2A	109.4
N1^ii^—Ni1—O2^iii^	93.06 (8)	N1—C2—H2B	109.4
O1^ii^—Ni1—O2^iii^	90.48 (7)	C1—C2—H2B	109.4
O1^i^—Ni1—O2^iii^	89.52 (7)	H2A—C2—H2B	108.0
O2—Ni1—O2^iii^	180.000 (1)	C2—N1—Ni1^iv^	119.67 (16)
O3—S1—O2	111.34 (11)	C2—N1—H1C	110 (2)
O3—S1—O1	112.85 (11)	Ni1^iv^—N1—H1C	108 (2)
O2—S1—O1	111.54 (11)	C2—N1—H1D	106 (2)
O3—S1—C1	106.05 (11)	Ni1^iv^—N1—H1D	107 (2)
O2—S1—C1	107.59 (12)	H1C—N1—H1D	106 (3)
O1—S1—C1	107.09 (11)		

Symmetry codes: (i) −*x*+1, −*y*+2, −*z*+2; (ii) *x*−1, *y*, *z*; (iii) −*x*, −*y*+2, −*z*+2; (iv) *x*+1, *y*, *z*.

### Hydrogen-bond geometry (Å, °)

**Table:**

*D*—H···*A*	*D*—H	H···*A*	*D*···*A*	*D*—H···*A*
N1—H1D···O3^v^	0.80 (3)	2.50 (3)	3.171 (3)	143 (3)
N1—H1C···O3^vi^	0.80 (3)	2.41 (3)	3.121 (3)	149 (3)

Symmetry codes: (v) −*x*+3/2, *y*−1/2, −*z*+3/2; (vi) *x*+1/2, −*y*+3/2, *z*+1/2.
